# Enhancing Email Responsiveness in Mental Health Services: A Quality Improvement Initiative

**DOI:** 10.1192/bjo.2025.10441

**Published:** 2025-06-20

**Authors:** Rakesh Sharma

**Affiliations:** CAMHS St Ann’s Hospital, London, United Kingdom

## Abstract

**Aims:** Effective communication is essential in mental health services, where timely decisions can significantly impact patient outcomes. Studies have shown that delayed responses to clinical communications can compromise patient safety, lead to treatment delays, and increase stress among professionals. The absence of standardized email response protocols within the North London Mental Health Trust has resulted in varying practices, with some professionals responding promptly while others may neglect emails, particularly those concerning patient care. This project seeks to address this inconsistency and improve the overall communication framework within the Trust.

Aims were to to develop and implement an email response protocol for mental health professionals in the North London Mental Health Trust, ensuring timely and consistent communication regarding patient care.

**Methods:** Co-Production: Engage stakeholders, including clients/carers, mental health professionals, managers, and admin representatives, to co-design the email response guidelines. Conduct surveys to gather insights on current practices and barriers to timely responses.

Identify and Understand: Conduct a baseline assessment of current email response times and practices using a survey distributed to all mental health professionals. Analyse the collected data to identify patterns of non-responsiveness and the reasons behind them (e.g. workload, lack of awareness of the importance of timely responses).

Develop Change Strategy: Based on stakeholder feedback and data analysis, draft clear email response guidelines that outline expected response times, prioritize urgent communications, and provide examples of acceptable email etiquette. Incorporate exceptions for individuals on leave, out of office, or in emergency situations.

**Results:** 1. Work-Related Email Volume:

The majority of mental health professionals receive between 20–50 emails per day, with a significant portion being patient-care related.

2. Response Time for Patient-Care Emails:

Around 60% of professionals respond to patient-related emails within 24 hours.

This increases to 80% when measured within 48 hours, indicating that while delays exist, the majority eventually respond within two days.

3. Factors Influencing Response Time:

High workload, email volume, and urgent clinical duties were cited as the most common barriers to quick responses.

Some professionals also noted a lack of standardized email response guidelines, leading to inconsistency in practices.

4. Challenges in Email Management:

Professionals expressed difficulty in managing high email volumes, particularly when dealing with urgent clinical responsibilities.

Lack of training on email prioritization was another reported challenge.

5. Recommendations from Professionals for Improvement:

Implementation of standardized email response guidelines.

Training on email management strategies and prioritization of urgent vs. non-urgent communications.

6. Impact on Patient Care and Communication:

A consensus among professionals was that delayed email responses negatively impact patient care, as they can lead to delayed decision-making and increased stress among teams.

7. Willingness to Engage in Further Training:

A majority of professionals were willing to participate in training related to improving email response times, indicating strong support for a structured intervention.

**Conclusion:** The implementation of a standardized email response protocol is vital for improving communication practices among mental health professionals in the North London Mental Health Trust. By addressing the current gaps in communication, we aim to enhance patient care and streamline inter-professional collaboration.

